# Effect of Vaccine-Elicited Antibodies on Colonization of Neisseria meningitidis Serogroup B and C Strains in a Human Bronchial Epithelial Cell Culture Model

**DOI:** 10.1128/CVI.00188-17

**Published:** 2017-10-05

**Authors:** Vianca Vianzon, Beate Illek, Gregory R. Moe

**Affiliations:** Center for Immunobiology and Vaccine Development, UCSF Benioff Children's Hospital Oakland Research Institute, Oakland, California, USA; Food and Drug Administration

**Keywords:** Neisseria meningitidis, capsular polysaccharide, colonization, vaccines

## Abstract

Capsular polysaccharide-protein conjugate vaccines protect individuals from invasive disease and decrease carriage, which reduces spread of the organism in the population. In contrast, antibodies elicited by plain polysaccharide or protein antigen-based meningococcal (Men) vaccines have little or no effect on decreasing carriage. In this study, we investigated the mechanism by which vaccine-induced human immunoglobulin G (IgG) antibodies affect colonization by meningococcal serogroup B (MenB) or C (MenC) strains using a human bronchial epithelial cell culture model (16HBE14o-). Fluorescence microscopy showed that bacteria colonizing the apical side of 16HBE14o- monolayers had decreased capsular polysaccharide on the bacterial surface that resulted from shedding the capsule and not decreased production of polysaccharide. Capsular polysaccharide shedding depended on the presence of 16HBE14o- cells and bacteria but not direct adherence of the bacteria to the cells. Treatment of bacteria and cells with postimmunization MenC-conjugate IgG or murine anti-MenB polysaccharide monoclonal antibodies (MAbs) inhibited capsule shedding, microcolony dispersal, and invasion of the 16HBE14o- cell monolayer. In contrast, the IgG responses elicited by immunization with MenC polysaccharide (PS), MenB outer membrane vesicle (OMV)-based, or factor H binding protein (FHbp)-based vaccines were not different than preimmune IgG or no-treatment response. The results provide new insights on the mechanism by which high-avidity anticapsular antibodies elicited by polysaccharide-conjugate vaccines affect meningococcal colonization. The data also suggest that any effect on colonization by IgG elicited by OMV- or FHbp-based vaccines may involve a different mechanism.

## INTRODUCTION

Neisseria meningitidis is a bacterial species that normally colonizes human upper airway epithelial cells. For reasons that are not completely understood (1), some strains move through the epithelial cell layer into the bloodstream, causing rapidly progressing bacteremia and meningitis, with relatively high rates of mortality and debilitating sequelae in survivors. Humans provide the only reservoir of meningococci, and transmission between individuals occurs through mucosal aerosols, with infants, children, and young adults having the highest rates of disease.

Like Haemophilus influenzae and Streptococcus pneumoniae, which can also cause bacteremia and meningitis, pathogenic meningococcal strains are encapsulated by polysaccharides. Strains producing meningococcal serogroup A, B, C, W, X, or Y capsular polysaccharides (MenABCWXY PS) cause most cases of meningococcal disease. Except for MenB PS, which is chemically similar to human polysialic acid, meningococcal capsular PSs are immunogenic, and vaccines containing them provide individual protection against disease (2). However, plain PS-based meningococcal vaccines have little effect on colonization and provide little or no protection against disease in the unvaccinated person through herd immunity. In contrast, as was first shown for Haemophilus influenzae capsular PS-protein conjugate vaccines (Hib) (3), linking capsular PS to proteins to provide T cell help (4) results in higher-avidity antibodies (5), immunologic memory (6), and longer-lived protection (7). In addition, as shown in several studies, population-wide use of Hib (8), MenA (9), and MenC (10) PS-conjugate vaccines provided herd protection by decreasing carriage and disease among both the vaccinated and unvaccinated. As a result, disease caused by these bacteria can be largely controlled at the population level.

Widespread use of meningococcal PS-conjugate vaccines against MenC or MenACYW has left MenB strains, for which there is no equivalent PS-conjugate vaccine, as the cause of a majority of meningococcal disease cases in North America and Europe (11). Strain-specific outer membrane vesicle (OMV) vaccines have been developed and used to stem outbreaks of MenB disease, but data on the effect of OMV vaccine-elicited antibodies on colonization are inconclusive or largely negative (12–15). Recently, vaccines containing neisserial human complement factor H binding protein (FHbp) have been licensed in the United States (16), and they provide much broader protection than OMV vaccines against MenB strains, as well as strains from other meningococcal capsular groups. The Pfizer vaccine (Trumenba, MenB-FHbp) contains two recombinant lipid-modified FHbp antigens, one each from two sequence variant subfamilies A and B. The GSK vaccine (Bexsero, MenB-4C) contains OMV and three recombinant protein antigens: FHbp from subfamily B, neisserial adhesin A (NadA), and neisserial heparin binding antigen (NHBA). Since the vaccines are relatively new and have not been used in large populations, little is known about their effects on meningococcal carriage and herd protection (17).

The control of meningococcal disease in large populations appears to depend mainly on the ability of antibodies elicited by capsular PS-protein conjugate vaccines to reduce carriage (18, 19). While several studies have described the overall effect of meningococcal PS-conjugate vaccines on carriage, little is known about the direct effects of the antibodies on colonizing bacteria. The purpose of this study was to investigate, mechanistically, the effects of IgG antibodies elicited by a MenC PS-conjugate vaccine on bacteria in a polarized airway epithelial cell model of meningococcal colonization compared to antibodies elicited by plain PS, OMV, and MenB-FHbp. In the following, we show that high-avidity IgG elicited by PS-protein conjugate vaccines was unique in affecting characteristics of colonizing MenB and MenC strains that limit the ability to cause disease and to disseminate between individuals.

## RESULTS

### Meningococcal 16HBE14o- colonization model.

To establish a model for the initial stage of meningococcal colonization with wild-type encapsulated MenB and MenC strains, we tested the ability of the bacteria to form colonies on Calu-3, CFBE41o-, H441, and 16HBE14o- airway epithelial cell lines. Bacteria were added to the apical surface of confluent cell monolayers on Transwell inserts and incubated in chemically defined cell culture medium containing human serum albumin under immersed culture conditions. CFBE41o- and 16HBE14o- cell lines form tight epithelial cell monolayers, which were confirmed by electrical resistance measurements and expression of zona occludens protein 1 (20). The bacteria in both the apical (top) and basolateral (bottom) sides of the chamber, as well as on the epithelial cell layer, were counted after 4 h, 8 h, and 16 h of incubation. Using a ratio of approximately ∼250 bacteria per 1 million epithelial cells as a reasonable approximation of human colonization, with respect to the number of epithelial cells being in large excess over the number of bacteria, 16HBE14o- cells gave the greatest and most consistent number of colonizing bacteria after 4 h of incubation (data not shown), which simulates the initial stage of meningococcal colonization. While invasion of the monolayer occurred as early as 4 h (MenC strain 4243), no bacteria were detected in the lower chamber up to 8 h after adding the bacteria.

Although secretory IgA elicited by immunization with meningococcal vaccines may have an important role in colonization, such antibodies were not available for our study. Alternatively, we used IgG, which is also present in mucosal secretions of vaccinated individuals (21). With anti-MenC PS IgG, we used amounts similar to those measured in mucosal secretions (21). The levels of IgG in the mucosa elicited by the protein-based meningococcal vaccines are not known. Therefore, we used amounts that are likely in excess of what would be expected from studies of capsular PS-protein conjugate vaccines as a best possible case test.

### Effect of vaccine-elicited IgG on meningococcal colonization of 16HBE14o- monolayers.

**FIG 1:**
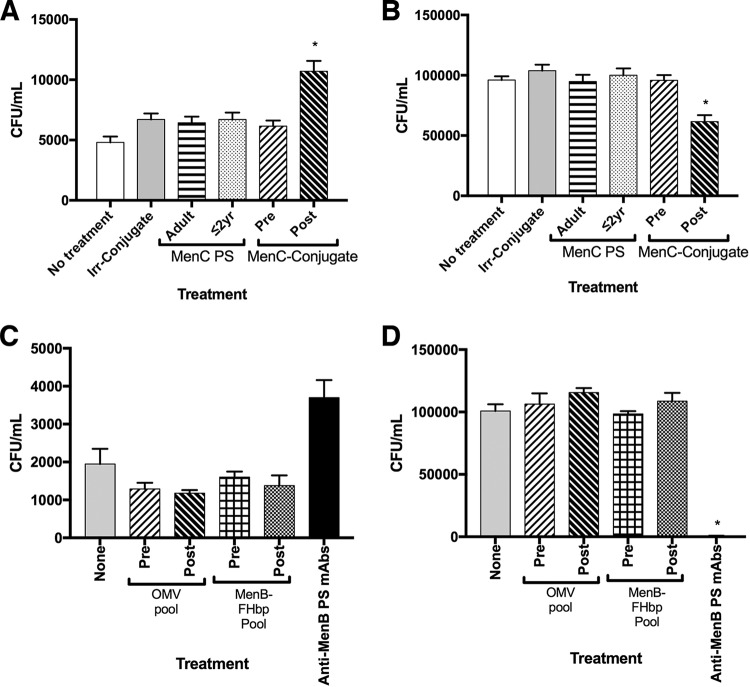
Effect of vaccine-elicited IgG treatment on the number of adherent and nonadherent bacteria cultured on the apical surface of 16HBE14o- cells. (A to D) Adherent (A) and nonadherent (B) MenC strain 4243, and adherent (C) and nonadherent (D) MenB strain H44/76. CFU per milliliter is the number of bacteria in the 200-μl trypsin solution. Pre and Post indicate preimmune and postimmune IgG, respectively. Each bar represents the mean ± standard error of the mean (SEM) of the results from 4 biological replicates. *, *P* < 0.05 compared to preimmune IgG or no treatment. The murine anti-MenB PS antibodies used to treat H44/76 were SEAM 2 and SEAM 12 (41). Only anticapsular antibodies elicited by PS-protein conjugate vaccines increased the number of adherent bacteria and decreased the number of nonadherent bacteria cultured on top of the 16HBE14o- cell monolayer compared to no-treatment or preimmune controls.

We purified IgG antibodies from pooled serum from children 9 months to 2 years old and adult subjects immunized with plain MenC PS vaccine (MenC PS), as well as adult subjects immunized with a MenC PS-conjugate vaccine (MenC-conjugate); then, we investigated the effect of IgG treatment on the adherence of MenC strain 4243 to 16HBE14o- cells during a 4-h incubation at 37°C. The controls included no treatment or treatment with IgG purified from serum from adults immunized with an irrelevant PS-conjugate vaccine (Irr-conjugate). Except for the no-treatment group, the amount of anti-MenC PS activity and total IgG concentration (adjusted with pre-MenC-conjugate IgG as nonimmune IgG when necessary) were the same for each treatment. As shown in Fig. 1A, the addition of post-MenC-conjugate IgG increased the number of CFU recovered from the 16HBE14o- monolayer, while the effects of the other IgG treatments were not significantly different from those of the no-treatment group. Conversely, the CFU in the apical fluid layer decreased with MenC-conjugate IgG treatment, whereas the CFU in all the other IgG treatments were no different than in the no-treatment group (Fig. 1B). The decreased CFU measured with MenC-conjugate IgG treatment did not appear to result from aggregates formed because of anticapsular antibody cross-linking of bacteria, since we did not observe any differences in the aggregation state of bacteria with anticapsular antibodies compared to no treatment or any treatment tested by microscopy or flow cytometry.

To determine the effect of purified IgG antibodies elicited by MenB-FHbp and OMV vaccines on colonization, we used MenB strain H44/76, which is a high expressor of FHbp, and our isolate of the strain used to prepare the OMV vaccine. The subfamily B FHbp antigen used in the MenB-FHbp vaccine is 93% identical to that of FHbp ID1 produced by H44/76, and IgG antibodies elicited by MenB-FHbp bind to H44/76, as determined by flow cytometry (data not shown). There were no significant differences in the number of colonizing bacteria treated with pooled pre- or post-OMV or MenB-FHbp IgG (Fig. 1C). Since MenB PS is chemically similar to human polysialic acid and is poorly immunogenic in humans, an equivalent pool of human anti-MenB PS IgG to compare with MenC-conjugate IgG was not available. However, treatment with a mixture of two murine anti-MenB PS monoclonal antibodies (MAbs) had a similar effect on MenB strain H44/76 as post-MenC-conjugate IgG had on MenC strain 4243, where the MAb treatment increased the number of adherent CFU and decreased CFU in the apical fluid (Fig. 1C and D, respectively). We also tested MenC strain 4243 treatment with post-MenB-FHbp IgG, but there were no effects on the number of adherent or apical solution bacteria compared to no-treatment or pre-MenC-conjugate controls (data not shown).

### Anticapsular antibodies inhibit shedding of capsular PS by MenC strain 4243 and MenB strain H44/76 colonizing 16HBE14o- cells.

**FIG 2:**
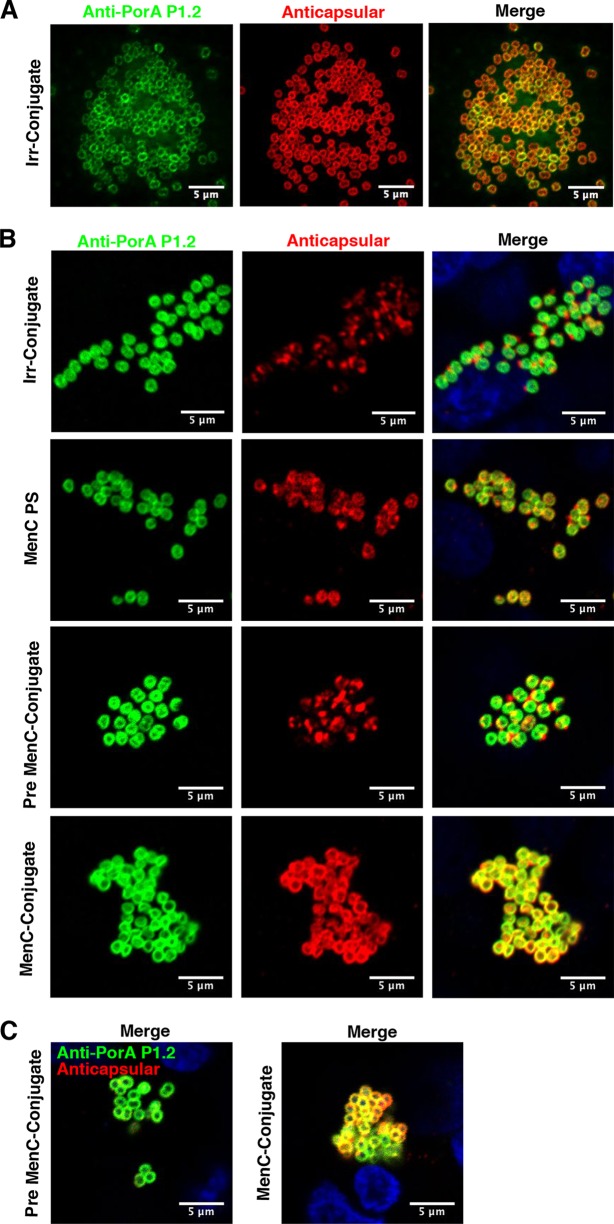
Fluorescence microscopy of MenC strain 4243 colonies. (A) Bacteria grown in cell culture medium in the absence of 16HBE14o- cells on coverslips. (B) Adherent bacteria colonizing 16HBE14o- cells after 4 h of incubation at 37°C and treated with the pool of purified IgG indicated on the left side of each row. (C) Adherent bacteria colonizing 16HBE14o- cells after 4 h of incubation at 32°C and treated with the pool of purified IgG indicated on the left of each panel. Blue, DAPI DNA stain; green, porin PorA P1.2; red, MenC capsular polysaccharide. Anticapsular antibodies elicited by MenC-conjugate promote the retention of capsular polysaccharide, which is otherwise shed in the presence of 16HBE14o- cells.

Previous studies indicated that meningococci invading epithelial cells are unencapsulated (22, 23); therefore, we investigated the effect of IgG treatments on MenC strain 4243 and MenB strain H44/76 capsular PS when grown in the presence of 16HBE14o- cells at 37°C using fluorescence microscopy. As a control, bacterial colonies grown in the absence of 16HBE14o- cells on poly-l-lysine-coated coverslips were fully encapsulated (Fig. 2A). In contrast, bacterial colonies attached to 16HBE14o- cells and treated with Irr-conjugate, adult MenC PS, or pre-MenC-conjugate IgG were less encapsulated (Fig. 2B), whereas bacteria treated with MenC-conjugate IgG retained capsular PS (Fig. 2B). Since lower temperature may decrease the production of capsular PS (24), we also looked at the MenC strain grown at 32°C in the presence of 16HBE14o- cells. As observed at the higher temperature, the bacteria were fully encapsulated when treated with post-MenC-conjugate IgG but almost completely unencapsulated in the presence of pre-MenC-conjugate IgG (Fig. 2C). The capsular polysaccharide remaining on relatively unencapsulated bacteria was concentrated in discrete areas and not colocalized with anti-porin PorA immunofluorescence, which marks the outer membrane (Fig. 2B). Also, particulate blebs of capsular polysaccharide shed from the bacteria were observed around colonies on the surface of the 16HBE14o- cells (data not shown). Taken together, the results show that the loss of capsular polysaccharide occurred by shedding rather than decreased production.

**FIG 3:**
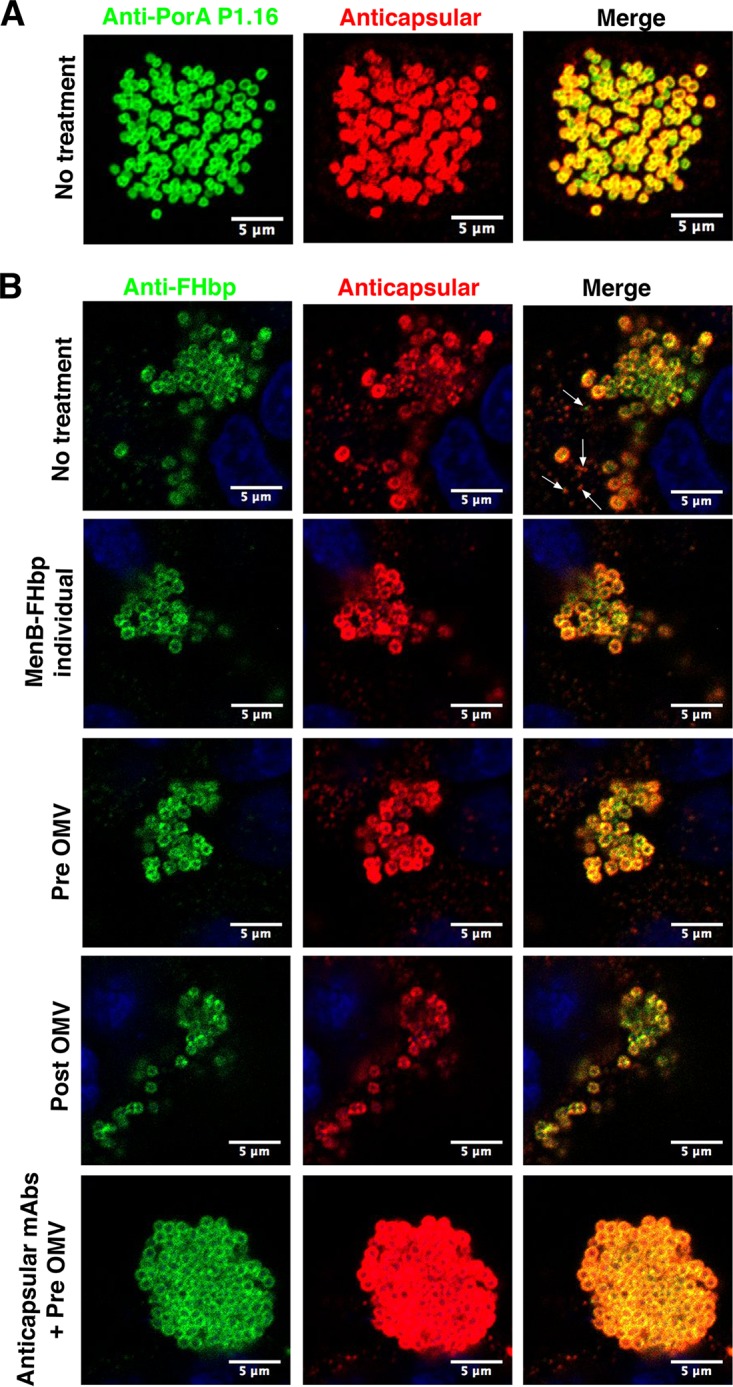
Fluorescence microscopy of MenB strain H44/76 colonies. (A) Bacteria grown in cell culture medium in the absence of 16HBE14o- cells on coverslips. (B) Adherent bacteria colonizing 16HBE14o- cells after 4 h of incubation at 37°C and treated with the pool of purified IgG or murine anti-MenB PS MAbs SEAM 2 and SEAM 12 (41), as indicated above each panel. Blue is DAPI DNA stain, green labels porin PorA P1.16 or FHbp, as indicated, and red labels MenB capsular PS. (B) Particles of shed capsular polysaccharide are indicated by arrows in the no-treatment control Merge panel. The anticapsular MAbs elicited by MenB PS conjugate vaccine (41) promoted the retention of capsular PS, which is otherwise shed in the presence of 16HBE14o- cells alone or with other IgG treatments.

Similarly, MenB strain H44/76 colonies were fully encapsulated when grown on poly-l-lysine-coated coverslips (Fig. 3A) but less encapsulated when cultured in the presence of 16HBE14o- cells alone or treated with MenB-FHbp IgG from an individual high-responder-as-best-case test or pre- or post-OMV IgG. As was the case with post-MenC-conjugate IgG, a mixture of two anticapsular MenB PS MAbs, combined with pre-OMV IgG as a control for the presence of human IgG (25), resulted in capsule retention (Fig. 3B). In contrast to the results with anti-PorA described above, staining marked by anti-FHbp murine MAb Jar5 was colocalized with capsular polysaccharide both on the bacterial membrane of MenB strain H44/76 and in blebs shed from the bacteria (see, for example, Fig. 3B, no treatment, indicated by arrows). The difference may reflect the fact that FHbp and capsular polysaccharide are associated with the outer membrane through lipid modifications, and there are potentially different mechanisms for the release of embedded versus associated outer membrane components during colonization.

### Loss of capsular polysaccharide in the presence of 16HBE14o- cells does not require adhesion to the cells.

**FIG 4:**
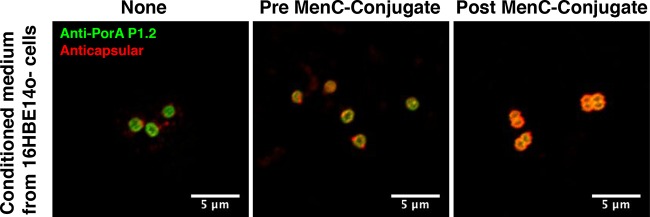
Effect of 16HBE14o- cells on capsular PS shedding by nonadherent bacteria. The images show nonadherent MenC strain 4243 bacteria in the medium from the apical fluid layer above 16HBE14o- cells after 4 h of incubation at 37°C with the indicated IgG treatment. The bacteria were fixed and labeled with anti-PorA P1.2 (green) and anti-MenC PS (red). The nonadherent bacteria shed capsular PS without treatment (None) or treatment with pooled pre-MenC-conjugate IgG (pre-MenC-conjugate), whereas capsular shedding was prevented by treatment with post-MenC-conjugate IgG (post-MenC-conjugate).

Next, we asked the question of whether the loss of capsular PS by MenC strain 4243 in the presence of 16HBE14o- cells required adhesion to the cells. After a 4-h incubation with 16HBE14o- cells, nonadherent bacteria in medium above the monolayer were collected and observed by fluorescence microscopy for the presence of capsular PS. As shown in Fig. 4, the nonadherent bacteria retained capsule in the presence of post- but not pre-MenC-conjugate IgG. However, conditioned medium collected from the apical solution above the 16HBE14o- monolayer after 4 h of incubation in the absence of bacteria did not induce shedding of capsular PS when the bacteria were grown for an additional 4 h in the absence of the 16HBE14o- cells (data not shown). Also, conditioning the medium with bacteria in the absence of 16HBE14o- cells, removing the bacteria, and growing a fresh inoculum of bacteria in the conditioned medium for 4 h did not result in capsular PS shedding. Therefore, the mechanism triggering the shedding of capsular PS requires the presence of both 16HBE14o- cells and bacteria but not direct adhesion of bacteria to the epithelial cell surface.

### Effect of vaccine-elicited IgG on invasion of 16HBE14o- cells.

**FIG 5:**
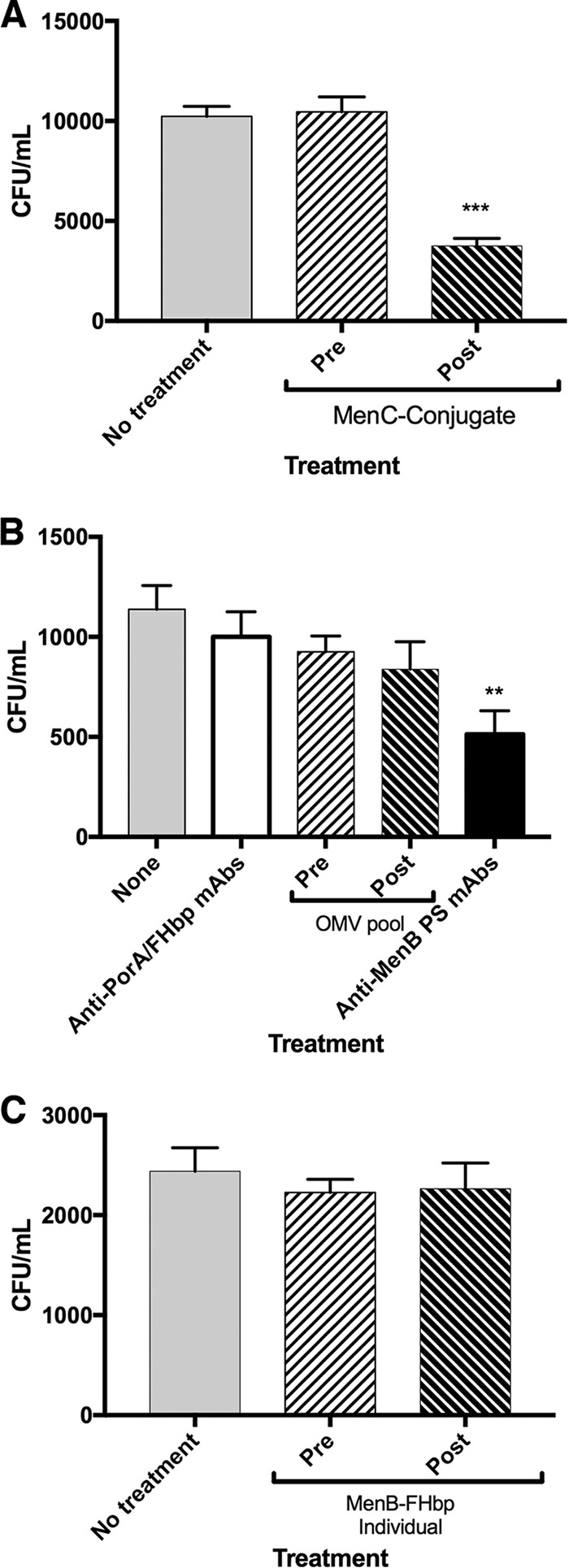
Effect of IgG on meningococcal invasion of 16HBE14o- cells by MenC strain 4243 (A) and MenB strain H44/76 (B and C). Invading bacteria were identified by resistance to gentamicin treatment. CFU per milliliter is the number of bacteria in the 200 μl of trypsin solution. The effects of no treatment and pre- and post-MenC-conjugate IgG were compared for MenC strain 4243 (A). The effects of no treatment or treatment with murine MAbs (anti-PorA P1.7, anti-FHbp Jar5, anticapsular SEAM 2, and SEAM 12), pre- and post-OMV IgG (B), and pre- and post-MenB-FHbp IgG from a single high responder (C) were compared for MenB strain H44/76. Each bar represents the mean ± SEM from 4 biological replicates. ***, *P* < 0.001 for postimmune compared to preimmune IgG or no treatment; **, *P* < 0.01 for anti-MenB PS MAbs compared to anti-PorA/FHbp MAbs. Only anticapsular IgG (post-MenC-conjugate and anticapsular MenB PS MAbs) elicited by PS-conjugate vaccines inhibited bacterial invasion of the 16HBE14o- cells.

Last, we investigated whether IgG treatments had an effect on the ability of meningococci to invade 16HBE14o- cells. Bacteria invading 16HBE14o- cells after 8 h of incubation at 37°C were detected by resistance to gentamicin treatment. Antibody treatments included no IgG (both strains), pre- or post-IgG from MenC-conjugate (MenC strain 4243), MenB-FHbp high-responding individual, and OMV (MenB strain H44/76) sera. Additional controls for strain H44/76 included mixtures of mouse anti-PorA P1.7 and anti-FHbp or anti-MenB PS MAbs. Intracellular bacteria were recovered for both strains (4243, Fig. 5A; H44/76, Fig. 5B and C). Only post-MenC-conjugate IgG and mouse anti-MenB PS MAb treatments significantly inhibited the invasion of MenC (Fig. 5A) and MenB (Fig. 5B).

## DISCUSSION

The introduction of PS-protein conjugate vaccines has had a profound effect on controlling disease caused by encapsulated bacterial pathogens in large populations. Among the most important benefits of these vaccines has been their ability to reduce carriage and disease through herd protection in unvaccinated individuals. While herd protection was demonstrated in many epidemiological studies (3, 8–10), the mechanisms by which PS-conjugate vaccine-elicited antibodies exert this effect remained unknown. The question of mechanism has become particularly important with the introduction of protein-based vaccines to protect against disease caused by MenB, whose capsular polysaccharide is similar to host polysialic acid, thus precluding the development of MenB PS-conjugate vaccines (26, 27). To address the question of mechanism, we characterized the effect of MenC-conjugate-elicited IgG compared to that with IgG elicited by other vaccines known to not affect colonization or provide herd protection or, in the case of the newly licensed “MenB vaccines” (16), to have unknown effects on colonization.

In the 16HBE14o- model, we observed that colonizing MenB and MenC strains shed capsule by blebbing capsular PS. Membrane blebbing has long been recognized as a characteristic of meningococcal strains that cause invasive disease (28). Also, studies of cellular invasion by meningococci in human tissue explants showed that bacteria invading epithelial cells lacked capsular PS, while neighboring noninvading bacteria bound to cilia retained capsule (22, 23). Several mechanisms have been described for the regulation of capsular PS production during epithelial cell invasion, including phase variation (29, 30), *misR-misS* two-component regulation of capsular operon gene expression (31), and temperature-dependent formation of mRNA hairpin structures that reduce expression of the polysialyltransferase at low temperature (24). However, in the 16HBE14o- model, the amount of capsular PS was not determined by regulating production but instead by shedding with MenC-conjugate IgG or anticapsular MenB PS MAbs inhibiting shedding. Shedding of the capsule did not depend on adhesion to the epithelial cell surface but required both 16HBE14o- cells and bacteria, suggesting the existence of an unknown soluble signaling molecule and bacterial system to regulate capsule shedding. Also, there may be differences in the mechanism of blebbing outer membrane containing integral membrane protein PorA versus lipid-modified capsular PS and FHbp, since blebs of lipid-modified capsular PS and FHbp were always colocalized by fluorescence microscopy, while blebs of PorA and capsular PS were not.

MenC-conjugate IgG and murine anti-MenB PS MAbs were unique among the vaccine-elicited IgG studied in the ability to prevent loss of capsule, increase the number of bacteria adherent to 16HBE14o- cells, decrease the number of bacteria in the fluid layer on the apical side of the cell monolayer, and inhibit invasion of the cell monolayer. Also, although we did not quantify the effect, treatment with PS-conjugate vaccine-elicited anticapsular antibodies resulted in adherent bacterial colonies that were qualitatively more condensed than no-IgG or other IgG treatments, suggesting that the high-avidity anticapsular antibodies inhibit microcolony dispersal as well (32). These effects may contribute to reducing carriage by making adherent encapsulated bacteria more efficiently killed by complement or cellular-dependent mechanisms or washed out by the flow of mucus in the airway. Also, reducing the number of bacteria in the fluid layer above the cell monolayer may have a similar effect of reducing bacteria in mucosal secretions that can be spread by aerosol droplets. It is unclear how anticapsular IgG blocks the loss of capsule, but we suggest that cross-linking of polysaccharide chains by high-avidity polyclonal IgG likely contributes to this effect. The ability of PS-conjugate vaccines to elicit high-avidity antibodies distinguishes these vaccines from plain PS, which elicits antibodies of generally lower avidity, particularly in infants (33).

In summary, this study provides new insights on how interactions of meningococcal strains with airway epithelial cells affect capsule shedding as a prerequisite to cellular invasion and inhibition of shedding by PS-conjugate vaccine-elicited IgG, as well as how these effects limit the potential for pathogenic meningococcal strains to spread among individuals. Given the unique effect of PS-conjugate vaccine-elicited IgG on capsule retention and characteristics of colonizing bacteria, it is clear that antibodies elicited by the existing recombinant FHbp protein- or OMV-based vaccines would affect carriage by a different mechanism, since they had no effect on capsule retention. Likely, protein vaccines will need to contain other antigens directly involved in colonization, such as conserved adhesins, in order to affect carriage and thus provide some measure of herd protection.

## MATERIALS AND METHODS

### Immunoglobulin G purification.

Donated human blood used in this study was obtained from donors under a protocol approved by the UCSF Benioff Children's Hospital Oakland institutional review board, with written informed consent obtained from all adult donor participants or from the legal guardians of the children who participated.

Pools were made from sera obtained from children 9 months to 2 years old (*n* = 7) and adults (*n* = 4) immunized with the ACYW quadrivalent PS (MenC PS) vaccine (34), sera from adults immunized with irrelevant pneumococcal 7-valent PS-conjugate vaccine (Prevnar) (*n* = 7) (35) (Irr-conjugate), and pre- and postvaccination sera from adults immunized with a MenC PS-CRM197 conjugate (MenC-conjugate) vaccine (*n* = 6) (36), outer membrane vesicle (OMV) vaccine (*n* = 5), and MenB-FHbp vaccine (*n* = 4). Pre- and postvaccination sera were also used from an individual who had a high response to the MenB-FHbp vaccine as a best-case test in some experiments. Immunoglobulin G (IgG) from the pooled sera was purified using separate 5-ml HiTrap protein G HP columns (GE Healthcare Bio-Sciences, Marlborough, MA, USA) on an Äkta fast-performance liquid chromatograph (FPLC; GE Healthcare Bio-Sciences). The IgG was eluted in 0.1 M histidine (pH 2.7) containing 0.02% (wt/vol) Tween 20 and immediately neutralized with 2 M Tris base. The solution was then dialyzed overnight in 4°C in 2 mM histidine (pH 6), 0.02% Tween 20, and 24 mM sucrose. Afterwards, it was lyophilized, resuspended in water to a concentration of 4 mg/ml, and then sterile-filtered.

For the pools of subjects immunized with MenC PS, MenC-conjugate, and Irr-conjugate vaccines, an enzyme-linked immunosorbent assay (ELISA) was done to determine the amounts of specific anti-MenC PS binding activity, as described previously (36). Binding activity was expressed in micrograms of MenC PS-specific IgG based on a comparison to human reference serum CDC1992 obtained from the National Institute for Biological Standards and Control, Hertfordshire, United Kingdom.

### Human bronchial epithelial cells.

The human epithelial cell line 16HBE14o- was originally obtained from a 1-year-old heart-lung transplant patient and immortalized with simian virus 40 (SV40) large T antigen by using the replication defective pSVori- plasmid (37). 16HBE14o- cells were kindly provided by D. C. Gruenert (University of California, San Francisco, CA) at passage 2.45 and cultured as previously described for transepithelial chloride ion transport measurements (38). For this study, cells were seeded on clear Transwell inserts (polyester membrane, 12-mm diameter, 0.4- μm or 3.0-μm pore size; Corning, Corning, NY, USA) that were previously coated with a mixture of fibronectin-vitrogen-bovine serum albumin in LHC basal medium (Thermo Fisher Scientific, Waltham, MA) (39, 40). Cells were plated at a density of 1 × 10^5^ cells/cm^2^ and grown to confluence in Eagle's minimal essential medium (Invitrogen, Carlsbad, CA, USA) supplemented with 10% fetal bovine serum (FBS; HyClone Characterized; GE Healthcare Bio-Sciences), 100 U/ml penicillin, 100 μg/ml streptomycin (UCSF Cell Culture Facility, San Francisco, CA, USA), and 2 mM glutamine (UCSF Cell Culture Facility). 16HBE14o- cell monolayers were grown under immerse culture conditions, with an apical volume of 500 μl and a basolateral volume of 1 ml to facilitate the formation of higher transepithelial electrical resistance values, as pointed out by Ehrhardt et al. (20). Cell monolayers were incubated in antibiotic-free medium (minimal essential medium [MEM], glutamine, and FBS) overnight, followed by two 30-min incubation periods with MEM, 4 mM d,l-lactate (Sigma-Aldrich, St. Louis, MO, USA), and 1 mg/ml human serum albumin (Sigma-Aldrich) before the start of each experiment.

### Colonization assay.

N. meningitidis strains were grown overnight on chocolate agar plates (Remel, Lenexa, KS) at 37°C with 5% CO_2_. Liquid cultures were then grown to mid-log phase in Frantz medium supplemented with 4 mM d,l-lactate. The bacteria were diluted 1:1,000,000 in MEM containing 1 mg/ml human serum albumin and 4 mM d,l-lactate. In each assay, the IgG treatments were adjusted to 0.05 μg of anti-MenC PS and 400 μg of total IgG with pre-MenC-conjugate IgG. The amount of MenC-conjugate IgG chosen for testing was based on preliminary experiments where the antibody was serially diluted and the effects on colonization (i.e., the number of bacteria in the medium described above and adhering to cells and the retention of capsular polysaccharide) were determined. We then used an amount that consistently produced the results shown in Fig. 1A and B and 2B of MenC-conjugate for each of the treatments. The IgG pools of subjects immunized with the OMV and MenB-FHbp vaccines were used at a final concentration of 2.5%. The amounts of MenB anticapsular MAbs, OMV, and MenB-FHbp IgG were arbitrary, since there was no effect on capsule retention, but the amounts were approximately 10× higher than serum bactericidal titers determined against the same strains with human complement. The amount of IgG was determined using the Bindarid human IgG RID kit (The Binding Site, Birmingham, UK). Bacteria were added to medium containing the antibody treatments in the Transwell inserts. After a 4-h incubation at 37°C with 5% CO_2_, the liquid above the cell monolayer was aspirated, the wells were washed twice with 500 μl of phosphate-buffered saline (PBS) containing 0.01% Tween 20, and 200 μl of 0.05% trypsin solution (UCSF Cell Culture Facility) was added. The plates were incubated on a shaker for 15 min at 37°C. The samples were then spotted on chocolate agar plates and tipped up to spread the solution. Viable CFU were counted after overnight incubation. Statistical comparison by two-tailed Mann-Whitney test was performed using GraphPad Prism version 7.0a for Mac OSX (GraphPad Software, Inc., San Diego, CA, USA).

### Conditioned medium.

Transwell inserts with 16HBE14o- cell monolayers were incubated for 4 h in medium (MEM, 1 mg/ml human serum albumin, and d,l-lactate) for conditioning. The liquid above the cell monolayer was collected, with a portion being filtered with a 10,000 molecular weight cutoff (MWCO) membrane (Corning). The conditioned media were then used for a 4-h incubation at 37°C with 5% CO_2_ with bacteria at a 1:1,000,000 dilution and various treatments on 24-well plates (Corning). The bacteria were harvested by centrifugation, suspended in 4% paraformaldehyde, and dried on coverslips. Afterwards, the coverslips were prepared for microscopy, as described below. Medium conditioned with the 16HBE14o- cell monolayer, which was infected with bacteria at a 1:1,000,000 dilution during the initial 4-h incubation and subsequently filtered, was also tested.

### Invasion assay.

After 8 h of incubation with or without IgG treatments, at 37°C with 5% CO_2_, the Transwell inserts with 16HBE14o- cell monolayers were washed twice with 500 μl of PBS with 0.01% Tween 20, followed by the addition of 200 μl of 200 μg/ml gentamicin (Thermo Fisher Scientific) in medium (MEM, 1 mg/ml human serum albumin, and d,l-lactate). After 1 h of incubation, the gentamicin was aspirated, the Transwell inserts were washed three times with 500 μl of Dulbecco's PBS (DPBS), and 200 μl of 0.05% trypsin was added. The plates were incubated on a shaker for 15 min at 37°C. The trypsin solution (10 μl) was spotted on chocolate agar plates and counted as described above. Statistical comparisons were made as described above.

### Microscopy.

Coverslips were coated with poly-l-lysine (Sigma-Aldrich) at a concentration of 1 mg/ml and dried at room temperature overnight before use. They were washed three times with sterile DPBS before adding bacteria. The liquid above the cell monolayer in Transwell inserts was aspirated after the 4-h incubation, the wells were washed twice with 500 μl of PBS and 0.01% Tween 20, and 500 μl of 4% paraformaldehyde was added. After a 20-min incubation, the liquid was aspirated, and 500 μl of blocking buffer (2% goat serum in DPBS and 0.02% Tween 20) was added for an overnight incubation at 4°C. Nonadherent bacteria from the colonization assay and bacteria from the conditioned medium assay were dried on plain coverslips after centrifugation and subsequent suspension using 4% paraformaldehyde. The coverslips were then blocked overnight as described above. Both the coverslips and the Transwell inserts were marked for PorA (P1.2 for 4243, P1.16 for H44/76), capsular PS (JW-C1 for 4243, SEAM 12 [41] for H44/76), factor H (sheep polyclonal antibody to factor H; Abcam, Cambridge, MA, USA), or factor H binding protein (Jar5), with detection using goat anti-mouse IgG Alexa Fluor 488 and goat anti-mouse IgG Alexa Fluor 594 subclass-specific secondary antibodies and 4′,6-diamidino-2-phenylindole (DAPI; Thermo Fisher Scientific) to stain the DNA. Images were recorded on a Zeiss LSM-710 confocal microscope (Zeiss, Oberkochen, Germany).
